# *N*′-(2,4-Dinitro­phen­yl)benzohydrazide

**DOI:** 10.1107/S1600536812030619

**Published:** 2012-07-10

**Authors:** Aamer Saeed, Ifzan Arshad, Ulrich Flörke

**Affiliations:** a Department of Chemistry, Quaid-i-Azam University, Islamabad 45320, Pakistan; b Department Chemie, Fakultät für Naturwissenschaften, Universität Paderborn, Warburgerstrasse 100, D-33098 Paderborn, Germany

## Abstract

In the title compound, C_13_H_10_N_4_O_5_, the aromatic ring planes are close to perpendicular [dihedral angle = 75.94 (5)°] and the C—N—N—C torsion angle is 88.7 (2)°. Both nitro groups lie close to their attached ring plane, with C—C—N—O torsion angles of 3.1 (2) and 5.3 (2)°. This allows for the formation of an intra­molecular N—H⋯O hydrogen bond, which closes an *S*(6) ring. In the crystal, N—H⋯O hydrogen bonds link the mol­ecules into zigzag chains extending along [100].

## Related literature

For a related structure, see: Wardell *et al.* (2007 ▶).
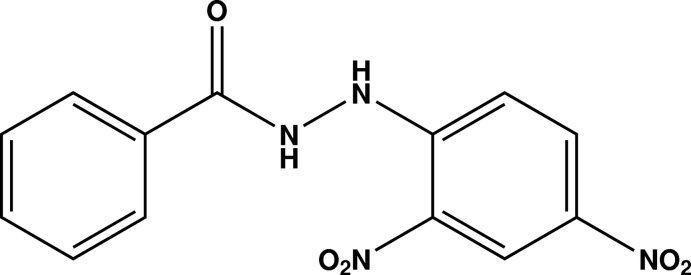


## Experimental

### 

#### Crystal data

C_13_H_10_N_4_O_5_
*M* *_r_* = 302.25
Monoclinic, 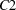

*a* = 13.5714 (10) Å
*b* = 8.4621 (6) Å
*c* = 11.4547 (9) Å
β = 93.830 (2)°
*V* = 1312.55 (17) Å^3^
*Z* = 4
Mo *K*α radiation
μ = 0.12 mm^−1^
*T* = 130 K
0.48 × 0.20 × 0.19 mm

#### Data collection

Bruker SMART APEX diffractometer
Absorption correction: multi-scan (*SADABS*; Sheldrick, 2004 ▶) *T*_min_ = 0.944, *T*_max_ = 0.977
6295 measured reflections
1673 independent reflections
1599 reflections with *I* > 2σ(*I*)
*R*_int_ = 0.018

#### Refinement

*R*[*F* ^2^ > 2σ(*F* ^2^)] = 0.030
*wR*(*F* ^2^) = 0.083
*S* = 1.06
1673 reflections
206 parameters
1 restraint
H atoms treated by a mixture of independent and constrained refinement
Δρ_max_ = 0.32 e Å^−3^
Δρ_min_ = −0.17 e Å^−3^

### 

Data collection: *SMART* (Bruker, 2002 ▶); cell refinement: *SAINT* (Bruker, 2002 ▶); data reduction: *SAINT*; program(s) used to solve structure: *SHELXTL* (Sheldrick, 2008 ▶); program(s) used to refine structure: *SHELXTL*; molecular graphics: *SHELXTL*; software used to prepare material for publication: *SHELXTL*.

## Supplementary Material

Crystal structure: contains datablock(s) global, I. DOI: 10.1107/S1600536812030619/hb6871sup1.cif

Structure factors: contains datablock(s) I. DOI: 10.1107/S1600536812030619/hb6871Isup2.hkl

Supplementary material file. DOI: 10.1107/S1600536812030619/hb6871Isup3.cml

Additional supplementary materials:  crystallographic information; 3D view; checkCIF report

## Figures and Tables

**Table 1:** Hydrogen-bond geometry (Å, °)

*D*—H⋯*A*	*D*—H	H⋯*A*	*D*⋯*A*	*D*—H⋯*A*
N1—H1⋯O1^i^	0.92 (3)	1.96 (3)	2.803 (2)	151 (2)
N2—H2⋯O3^ii^	0.81 (3)	2.30 (2)	2.968 (2)	140 (2)
N2—H2⋯O3	0.81 (3)	2.02 (2)	2.606 (2)	129 (2)

Symmetry codes: (i) 
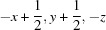
; (ii) 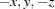.

**Fig. 1.**
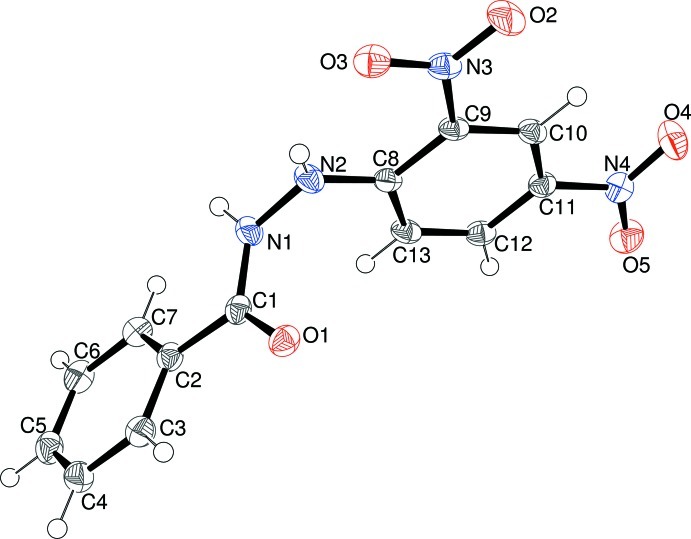
Molecular structure of the title compound. Anisotropic displacement ellipsoids drawn at the 50% probability level.

**Fig. 2.**
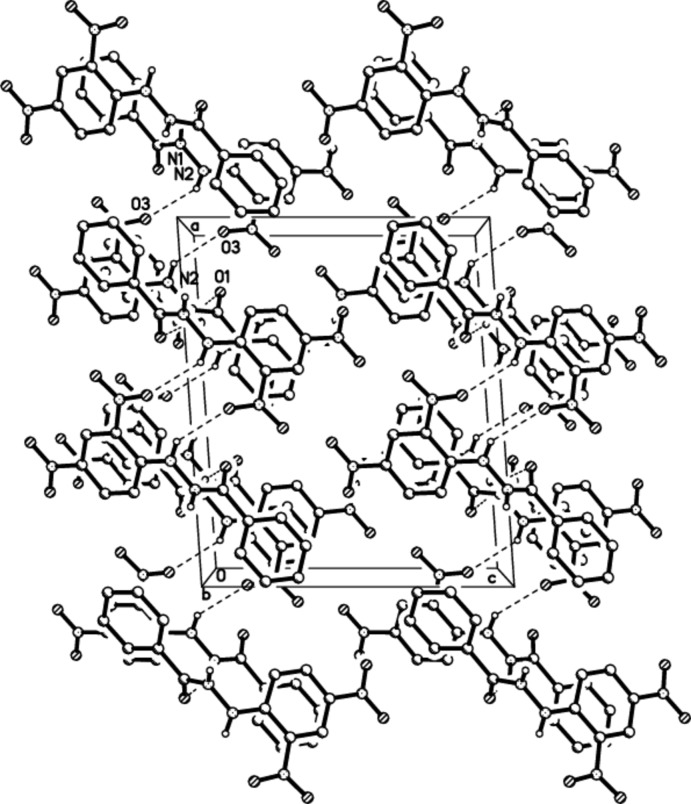
Crystal packing viewd along a-axis with intermolecular hydrogen bonding pattern as dashed lines. H atoms not involved are omitted.

## supplementary crystallographic information

### Comment

The PhC(O)NNPh core moiety of the title compound (Figure 1) is similar to that of N-anilino-4-nitrobenzamide (Wardell *et al.*, 2007) with different ring substituents. The molecular conformation is determined by an intra-molecular N2–H···O3 hydrogen bond with H···O3 2.02 (2) Å and an associated torsion angle N2–C8–C9–N3 of -0.1 (3)°. The inter-molecular hydrogen bonds N1–H···O1(-x+0.5, y+0.5, -z) with H···O 1.96 (3) Å and N-H···O 151 (2)° as well as N2–H···O3(-x, y, -z) with H···O 2.30 (2) Å and C-H···O 140 (2)° link molecules into zigzag chains extended along the a-axis (Figure 2).

### Experimental

2,4-Dinitrophenyl hydrazine (2.8 mmol) in dry CH_2_Cl_2_ was treated with benzoyl chloride (2.8 mmol) and the mixture was refluxed for 3 hours. On completion of reaction, the mixture was allowed to cool and excess of solvent was evaporated under reduced pressure. Yellow prisms of the title compound were recrystallized from ethanol solution by slow evaporation of the solvent at room temperature (m.p 213-215°C).

### Refinement

Hydrogen atoms were clearly identified in difference syntheses, refined at idealized positions riding on the carbon atoms with isotropic displacement parameters Uiso(H) = 1.2U(C_eq_) and C—H 0.95 Å. H(N) atoms were refined freely. The title compound crystallizes in the non-centrosymmetric space group C 2; however, in the absence of significant anomalous scattering effects, Friedel pairs were merged.

### Crystal data

**Table:**

C_13_H_10_N_4_O_5_	*F*(000) = 624
*M**_r_* = 302.25	*D*_x_ = 1.530 Mg m^−^^3^
Monoclinic, *C*2	Mo *K*α radiation, λ = 0.71073 Å
*a* = 13.5714 (10) Å	Cell parameters from 2900 reflections
*b* = 8.4621 (6) Å	θ = 2.8–28.1°
*c* = 11.4547 (9) Å	µ = 0.12 mm^−^^1^
β = 93.830 (2)°	*T* = 130 K
*V* = 1312.55 (17) Å^3^	Prism, yellow
*Z* = 4	0.48 × 0.20 × 0.19 mm

### Data collection

**Table:**

Bruker SMART APEX diffractometer	1673 independent reflections
Radiation source: sealed tube	1599 reflections with *I* > 2σ(*I*)
Graphite monochromator	*R*_int_ = 0.018
φ and ω scans	θ_max_ = 27.9°, θ_min_ = 1.8°
Absorption correction: multi-scan (*SADABS*; Sheldrick, 2004)	*h* = −17→17
*T*_min_ = 0.944, *T*_max_ = 0.977	*k* = −11→10
6295 measured reflections	*l* = −15→15

### Refinement

**Table:**

Refinement on *F*^2^	Primary atom site location: structure-invariant direct methods
Least-squares matrix: full	Secondary atom site location: difference Fourier map
*R*[*F*^2^ > 2σ(*F*^2^)] = 0.030	Hydrogen site location: difference Fourier map
*wR*(*F*^2^) = 0.083	H atoms treated by a mixture of independent and constrained refinement
*S* = 1.06	*w* = 1/[σ^2^(*F*_o_^2^) + (0.055*P*)^2^ + 0.2958*P*] where *P* = (*F*_o_^2^ + 2*F*_c_^2^)/3
1673 reflections	(Δ/σ)_max_ < 0.001
206 parameters	Δρ_max_ = 0.32 e Å^−^^3^
1 restraint	Δρ_min_ = −0.17 e Å^−^^3^

### Special details

**Table:**

Geometry. All esds (except the esd in the dihedral angle between two l.s. planes) are estimated using the full covariance matrix. The cell esds are taken into account individually in the estimation of esds in distances, angles and torsion angles; correlations between esds in cell parameters are only used when they are defined by crystal symmetry. An approximate (isotropic) treatment of cell esds is used for estimating esds involving l.s. planes.
Refinement. Refinement of F^2^ against ALL reflections. The weighted R-factor wR and goodness of fit S are based on F^2^, conventional R-factors R are based on F, with F set to zero for negative F^2^. The threshold expression of F^2^ > 2sigma(F^2^) is used only for calculating R-factors(gt) etc. and is not relevant to the choice of reflections for refinement. R-factors based on F^2^ are statistically about twice as large as those based on F, and R- factors based on ALL data will be even larger.

### Fractional atomic coordinates and isotropic or equivalent isotropic displacement parameters (Å^2^)

**Table:**

	*x*	*y*	*z*	*U*_iso_*/*U*_eq_	
O1	0.17978 (9)	0.28363 (16)	−0.09274 (11)	0.0246 (3)	
O2	−0.07017 (10)	0.5010 (2)	0.29368 (13)	0.0372 (4)	
O3	−0.01782 (10)	0.57932 (19)	0.12955 (11)	0.0279 (3)	
O4	0.11363 (10)	0.1665 (2)	0.55486 (12)	0.0357 (4)	
O5	0.25511 (12)	0.0749 (2)	0.51048 (13)	0.0336 (3)	
N1	0.23735 (12)	0.5032 (2)	0.00043 (13)	0.0236 (3)	
H1	0.2805 (17)	0.586 (3)	0.0130 (19)	0.027 (4)*	
N2	0.15908 (12)	0.5061 (2)	0.07220 (13)	0.0236 (3)	
H2	0.1069 (18)	0.542 (3)	0.0466 (19)	0.027 (4)*	
N3	−0.00676 (11)	0.50411 (19)	0.22250 (13)	0.0236 (3)	
N4	0.18149 (11)	0.1576 (2)	0.48955 (13)	0.0251 (3)	
C1	0.24091 (12)	0.3905 (2)	−0.08297 (14)	0.0205 (3)	
C2	0.32573 (13)	0.4038 (2)	−0.15959 (15)	0.0211 (3)	
C3	0.32016 (14)	0.3169 (2)	−0.26364 (15)	0.0245 (4)	
H3A	0.2639	0.2531	−0.2835	0.029*	
C4	0.39670 (14)	0.3239 (3)	−0.33789 (16)	0.0270 (4)	
H4A	0.3925	0.2654	−0.4088	0.032*	
C5	0.47950 (14)	0.4162 (2)	−0.30909 (17)	0.0278 (4)	
H5A	0.5319	0.4204	−0.3601	0.033*	
C6	0.48556 (14)	0.5025 (3)	−0.20550 (17)	0.0278 (4)	
H6A	0.5422	0.5655	−0.1858	0.033*	
C7	0.40929 (13)	0.4967 (2)	−0.13092 (15)	0.0245 (4)	
H7A	0.4137	0.5558	−0.0603	0.029*	
C8	0.16356 (12)	0.4225 (2)	0.17329 (14)	0.0193 (3)	
C9	0.08447 (12)	0.4188 (2)	0.24836 (15)	0.0196 (3)	
C10	0.09033 (13)	0.3322 (2)	0.35177 (15)	0.0208 (3)	
H10A	0.0366	0.3305	0.4008	0.025*	
C11	0.17514 (12)	0.2492 (2)	0.38177 (15)	0.0210 (3)	
C12	0.25477 (13)	0.2501 (2)	0.31123 (15)	0.0221 (4)	
H12A	0.3128	0.1915	0.3334	0.027*	
C13	0.24890 (12)	0.3360 (2)	0.20964 (15)	0.0222 (4)	
H13A	0.3038	0.3373	0.1624	0.027*	

### Atomic displacement parameters (Å^2^)

**Table:**

	*U*^11^	*U*^22^	*U*^33^	*U*^12^	*U*^13^	*U*^23^
O1	0.0219 (6)	0.0214 (6)	0.0306 (6)	−0.0018 (5)	0.0028 (5)	0.0007 (5)
O2	0.0236 (7)	0.0448 (9)	0.0449 (8)	0.0141 (7)	0.0145 (6)	0.0069 (7)
O3	0.0240 (6)	0.0297 (7)	0.0294 (6)	0.0053 (6)	−0.0025 (5)	−0.0004 (6)
O4	0.0279 (7)	0.0482 (10)	0.0319 (7)	0.0008 (7)	0.0084 (5)	0.0095 (7)
O5	0.0320 (7)	0.0326 (8)	0.0354 (7)	0.0077 (7)	−0.0031 (5)	0.0069 (6)
N1	0.0225 (7)	0.0249 (8)	0.0243 (6)	−0.0025 (7)	0.0078 (6)	−0.0003 (6)
N2	0.0185 (7)	0.0284 (8)	0.0244 (7)	0.0034 (7)	0.0054 (6)	0.0001 (7)
N3	0.0183 (7)	0.0231 (8)	0.0296 (7)	0.0032 (6)	0.0020 (5)	−0.0042 (6)
N4	0.0237 (7)	0.0252 (8)	0.0261 (7)	−0.0012 (6)	−0.0006 (6)	0.0015 (7)
C1	0.0187 (8)	0.0204 (8)	0.0223 (7)	0.0026 (7)	0.0001 (6)	0.0051 (6)
C2	0.0199 (8)	0.0206 (8)	0.0227 (8)	0.0022 (7)	0.0016 (6)	0.0039 (7)
C3	0.0247 (9)	0.0234 (9)	0.0255 (8)	−0.0012 (7)	0.0013 (6)	0.0005 (7)
C4	0.0293 (9)	0.0271 (9)	0.0248 (8)	0.0039 (8)	0.0040 (7)	0.0024 (7)
C5	0.0246 (9)	0.0285 (10)	0.0311 (9)	0.0041 (8)	0.0079 (7)	0.0077 (8)
C6	0.0211 (8)	0.0269 (10)	0.0353 (9)	−0.0004 (8)	0.0013 (7)	0.0052 (8)
C7	0.0231 (8)	0.0241 (9)	0.0261 (8)	0.0000 (8)	−0.0002 (6)	0.0003 (7)
C8	0.0174 (8)	0.0180 (8)	0.0228 (8)	0.0000 (7)	0.0033 (6)	−0.0036 (7)
C9	0.0145 (7)	0.0192 (8)	0.0252 (8)	0.0017 (6)	0.0021 (6)	−0.0042 (7)
C10	0.0162 (7)	0.0217 (8)	0.0250 (8)	−0.0005 (7)	0.0047 (6)	−0.0040 (7)
C11	0.0209 (8)	0.0189 (8)	0.0230 (7)	−0.0009 (7)	0.0011 (6)	−0.0024 (6)
C12	0.0176 (8)	0.0212 (9)	0.0273 (8)	0.0022 (7)	0.0005 (6)	−0.0036 (7)
C13	0.0161 (7)	0.0239 (9)	0.0269 (8)	0.0006 (7)	0.0049 (6)	−0.0057 (7)

### Geometric parameters (Å, º)

**Table:**

O1—C1	1.227 (2)	C4—C5	1.390 (3)
O2—N3	1.2247 (19)	C4—H4A	0.9500
O3—N3	1.241 (2)	C5—C6	1.391 (3)
O4—N4	1.2271 (19)	C5—H5A	0.9500
O5—N4	1.230 (2)	C6—C7	1.386 (2)
N1—C1	1.353 (2)	C6—H6A	0.9500
N1—N2	1.386 (2)	C7—H7A	0.9500
N1—H1	0.92 (3)	C8—C13	1.409 (2)
N2—C8	1.355 (2)	C8—C9	1.420 (2)
N2—H2	0.81 (3)	C9—C10	1.391 (2)
N3—C9	1.447 (2)	C10—C11	1.372 (2)
N4—C11	1.455 (2)	C10—H10A	0.9500
C1—C2	1.498 (2)	C11—C12	1.392 (2)
C2—C3	1.398 (2)	C12—C13	1.370 (3)
C2—C7	1.401 (3)	C12—H12A	0.9500
C3—C4	1.387 (2)	C13—H13A	0.9500
C3—H3A	0.9500		
			
C1—N1—N2	119.72 (16)	C6—C5—H5A	120.1
C1—N1—H1	126.7 (14)	C7—C6—C5	120.21 (17)
N2—N1—H1	113.5 (14)	C7—C6—H6A	119.9
C8—N2—N1	120.36 (15)	C5—C6—H6A	119.9
C8—N2—H2	119.7 (16)	C6—C7—C2	120.09 (17)
N1—N2—H2	118.6 (15)	C6—C7—H7A	120.0
O2—N3—O3	122.17 (14)	C2—C7—H7A	120.0
O2—N3—C9	118.85 (15)	N2—C8—C13	120.84 (15)
O3—N3—C9	118.98 (13)	N2—C8—C9	122.46 (15)
O4—N4—O5	123.30 (16)	C13—C8—C9	116.69 (15)
O4—N4—C11	118.70 (15)	C10—C9—C8	121.68 (15)
O5—N4—C11	118.00 (15)	C10—C9—N3	115.90 (14)
O1—C1—N1	121.88 (15)	C8—C9—N3	122.42 (15)
O1—C1—C2	122.90 (16)	C11—C10—C9	118.82 (15)
N1—C1—C2	115.21 (15)	C11—C10—H10A	120.6
C3—C2—C7	119.45 (16)	C9—C10—H10A	120.6
C3—C2—C1	117.41 (15)	C10—C11—C12	121.51 (16)
C7—C2—C1	123.14 (15)	C10—C11—N4	119.08 (15)
C4—C3—C2	120.02 (17)	C12—C11—N4	119.41 (15)
C4—C3—H3A	120.0	C13—C12—C11	119.51 (16)
C2—C3—H3A	120.0	C13—C12—H12A	120.2
C3—C4—C5	120.34 (18)	C11—C12—H12A	120.2
C3—C4—H4A	119.8	C12—C13—C8	121.78 (15)
C5—C4—H4A	119.8	C12—C13—H13A	119.1
C4—C5—C6	119.88 (17)	C8—C13—H13A	119.1
C4—C5—H5A	120.1		
			
C1—N1—N2—C8	88.7 (2)	N2—C8—C9—N3	−0.1 (3)
N2—N1—C1—O1	−4.6 (3)	C13—C8—C9—N3	179.06 (16)
N2—N1—C1—C2	176.85 (15)	O2—N3—C9—C10	3.1 (2)
O1—C1—C2—C3	17.0 (3)	O3—N3—C9—C10	−177.61 (16)
N1—C1—C2—C3	−164.49 (16)	O2—N3—C9—C8	−176.91 (17)
O1—C1—C2—C7	−162.30 (17)	O3—N3—C9—C8	2.4 (2)
N1—C1—C2—C7	16.2 (2)	C8—C9—C10—C11	0.3 (3)
C7—C2—C3—C4	−0.4 (3)	N3—C9—C10—C11	−179.65 (16)
C1—C2—C3—C4	−179.70 (16)	C9—C10—C11—C12	0.0 (3)
C2—C3—C4—C5	0.4 (3)	C9—C10—C11—N4	−179.65 (16)
C3—C4—C5—C6	−0.2 (3)	O4—N4—C11—C10	−5.3 (2)
C4—C5—C6—C7	0.0 (3)	O5—N4—C11—C10	174.74 (18)
C5—C6—C7—C2	0.1 (3)	O4—N4—C11—C12	175.03 (17)
C3—C2—C7—C6	0.1 (3)	O5—N4—C11—C12	−4.9 (2)
C1—C2—C7—C6	179.42 (17)	C10—C11—C12—C13	0.2 (3)
N1—N2—C8—C13	2.4 (3)	N4—C11—C12—C13	179.91 (16)
N1—N2—C8—C9	−178.54 (15)	C11—C12—C13—C8	−0.9 (3)
N2—C8—C9—C10	179.94 (17)	N2—C8—C13—C12	−179.65 (17)
C13—C8—C9—C10	−0.9 (2)	C9—C8—C13—C12	1.2 (3)

### Hydrogen-bond geometry (Å, º)

**Table:**

*D*—H···*A*	*D*—H	H···*A*	*D*···*A*	*D*—H···*A*
N1—H1···O1^i^	0.92 (3)	1.96 (3)	2.803 (2)	151 (2)
N2—H2···O3^ii^	0.81 (3)	2.30 (2)	2.968 (2)	140 (2)
N2—H2···O3	0.81 (3)	2.02 (2)	2.606 (2)	129 (2)

Symmetry codes: (i) −*x*+1/2, *y*+1/2, −*z*; (ii) −*x*, *y*, −*z*.
